# A Delayed Sterile Abscess Following a Percutaneous Coronary Intervention (PCI)-Related Myocardial Injury Successfully Treated With an Omental Flap: A Case Report

**DOI:** 10.7759/cureus.86890

**Published:** 2025-06-27

**Authors:** Shunya Ono, Kazuki Morooka, Motoharu Shimozawa, Kosaku Nishigawa, Takeyuki Kanemura

**Affiliations:** 1 Cardiovascular Surgery, IMS Katsushika Heart Center, Tokyo, JPN

**Keywords:** hemostatic material, hydrofit®, omental flap, sterile abscess, tachosil®

## Abstract

Topical hemostatic agents are commonly used in cardiac surgery to achieve rapid hemostasis in fragile myocardial tissue, particularly following myocardial infarction or mechanical injury during percutaneous coronary intervention (PCI). While effective in the acute setting, retained hemostatic materials can lead to delayed complications, including sterile abscess formation.

We present a case of a 77-year-old man who developed a sterile abscess one year and nine months after emergency surgical hemostasis for myocardial oozing following PCI. The abscess presented as localized swelling and discharge from the lower end of the sternotomy scar. Laboratory tests showed no signs of systemic inflammation, and cultures were negative. Computed tomography (CT) revealed a fluid collection extending from the pericardial space to the subcutaneous tissue. Conservative treatment, including antibiotics and drainage, was ineffective. Surgical removal of the foreign material was performed under cardiopulmonary bypass. Due to dense adhesions and uncertainty regarding complete removal, a pedicled omental flap was transposed through the diaphragm into the pericardial space. Postoperative recovery was uneventful, and follow-up imaging confirmed complete resolution of the fluid collection. This case highlights the diagnostic challenge of sterile abscesses related to retained surgical materials and demonstrates the utility of omental transposition when complete removal is not feasible.

## Introduction

Hemostatic agents are commonly used in cardiovascular surgery to control bleeding in fragile myocardial tissues, especially following myocardial infarction or mechanical injury during percutaneous coronary intervention (PCI) [1-3]. These agents offer rapid hemostasis and reduce the need for complex suturing in vulnerable areas, making them indispensable tools in modern cardiac surgery.

Despite their hemostatic efficacy, the long-term safety of these materials remains a matter of concern. Foreign body reactions, sterile abscess formation, and late-onset mediastinal complications have been increasingly reported in association with retained hemostatic agents [4,5]. While postoperative infections following cardiac surgery are not uncommon, sterile abscesses arising from retained hemostatic material, without microbial involvement, represent a distinct and often underrecognized clinical entity [6]. These sterile collections may mimic infectious processes but are characterized by negative cultures, absence of systemic inflammatory response, and a chronic, indolent course [5].

In this report, we present a rare case of a sterile abscess occurring one year and nine months after emergency surgical hemostasis for myocardial oozing following PCI. The abscess was found to originate from retained hemostatic materials and was successfully treated via surgical excision and transdiaphragmatic placement of a pedicled omental flap. The case highlights diagnostic and therapeutic challenges and emphasizes the potential utility of omental transposition in managing non-infectious mediastinal complications.

## Case presentation

A 77-year-old man with a medical history of well-controlled hypertension, type 2 diabetes mellitus, and atrial fibrillation - treated with catheter ablation and currently maintaining sinus rhythm - underwent diagnostic coronary angiography due to exertional chest pain, which revealed significant stenosis in the left anterior descending (LAD) artery. The patient subsequently underwent PCI for the LAD lesion.

Five hours post-procedure, the patient developed hypotension, with a systolic blood pressure in the 60 mmHg range, and complained of chest discomfort. Urgent transthoracic echocardiography demonstrated significant pericardial effusion suggestive of cardiac tamponade. The patient was immediately transferred to the operating room for emergency surgical intervention via median sternotomy. Intraoperatively, persistent oozing-type bleeding was identified from the epicardial surface of the myocardium in the region of the diagonal branch. This was presumed to have resulted from mechanical perforation by the guidewire during the preceding PCI. As the bleeding appeared to stem from a localized area without frank myocardial tearing, initial hemostasis was achieved using a mattress suture reinforced with Teflon felt. However, due to the partial friability of the surrounding myocardium - likely reflecting underlying ischemic changes - additional topical hemostatic agents, including TachoSil® (Nycomed, Linz, Austria) and Hydrofit® (Sanyo Chemical Industries, Kyoto, Japan), were applied to ensure secure and atraumatic hemostasis. The postoperative course was uneventful, and the patient was discharged after an appropriate recovery.

**Figure 1:**
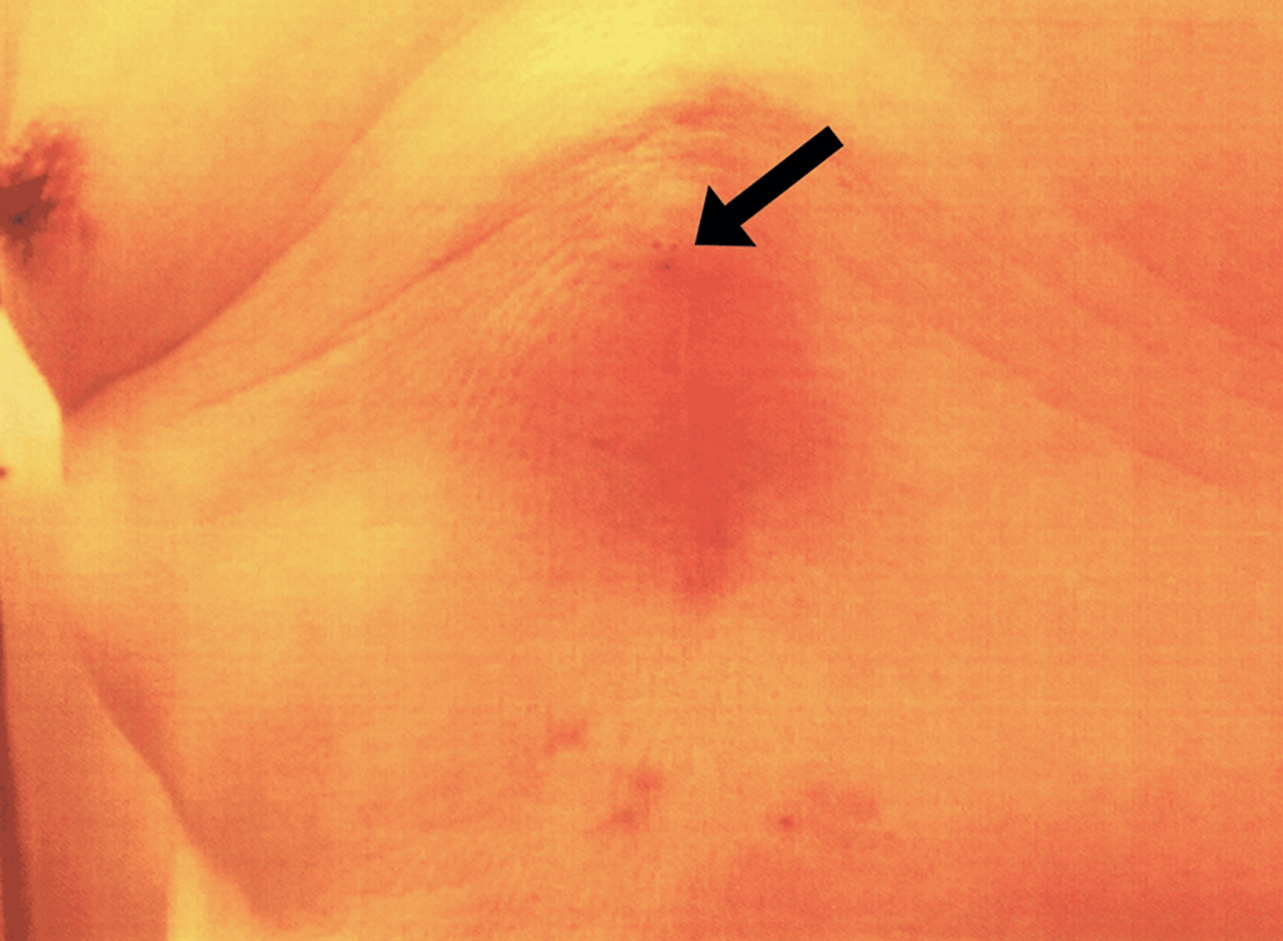
Clinical photograph of the surgical site Localized redness and swelling are observed at the lower end of the median sternotomy wound. Occasional discharge of yellowish, turbid pus was noted from the site indicated by the black arrow.

The patient remained clinically stable during regular outpatient follow-up. However, one year and nine months after the initial surgery, the patient presented with swelling, redness, and intermittent purulent discharge at the lower end of his median sternotomy scar (Figure 1).

The patient denied systemic symptoms such as fever or malaise. Physical examination revealed a localized area of fluctuance and erythema, without signs of systemic infection.

**Figure 2:**
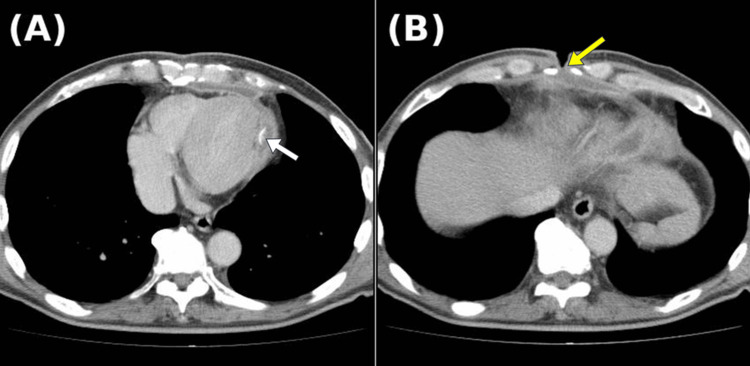
Preoperative computed tomography (CT) images (A) Residual Teflon felt is observed on the lateral wall of the left ventricle (white arrow), with encapsulated fluid extending along the anterior chest wall. (B) Communication between the subcutaneous incision site and the encapsulated fluid collection is visualized (yellow arrow).

Laboratory investigations showed no leukocytosis or elevation of inflammatory markers. Wound cultures were negative for bacterial growth. Contrast-enhanced computed tomography (CT) of the chest revealed a low-density, encapsulated fluid collection extending from the anterior chest wall to the area of the prior myocardial hemostasis (Figure 2A). This fluid collection was continuous with the surgical wound, indicating a direct tract between the intrathoracic space and the subcutaneous tissue (Figure 2B).

**Figure 3:**
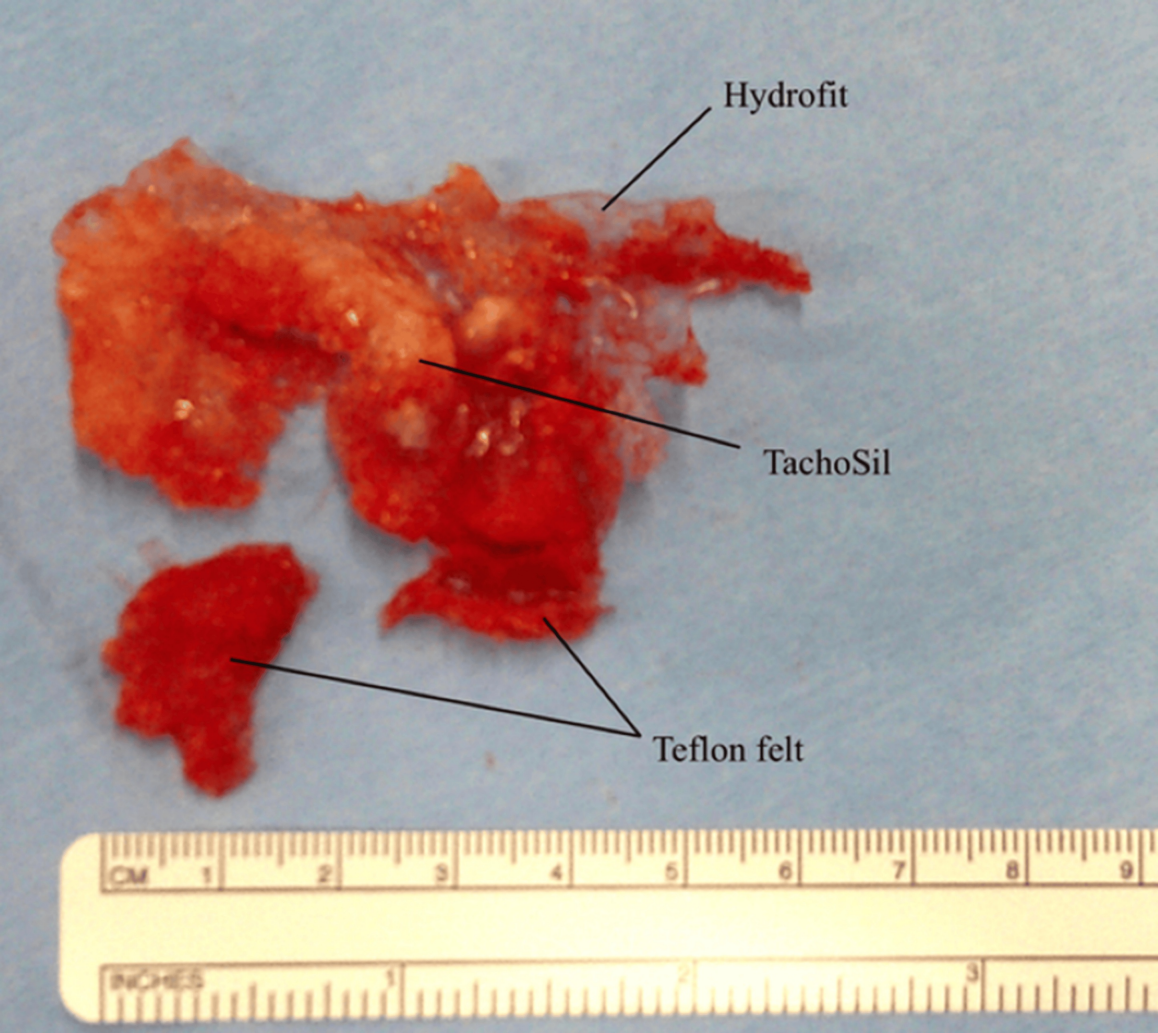
Foreign materials removed during surgery Residual bioabsorbable TachoSil is also identified.

Initial management included empirical oral antibiotics with cefaclor and percutaneous drainage. However, these measures failed to resolve the drainage or reduce the subcutaneous collection. Surgical intervention was elected for definitive management. Under general anesthesia, the previous sternotomy was reopened. Dense adhesions were encountered, and cardiopulmonary bypass was established via femoral cannulation due to the high risk of cardiac injury during dissection. Adhesions were carefully separated while the heart was still beating. All visible foreign materials, including TachoSil remnants, Hydrofit, and Teflon felt, were excised to the extent safely possible (Figure 3).

**Figure 4:**
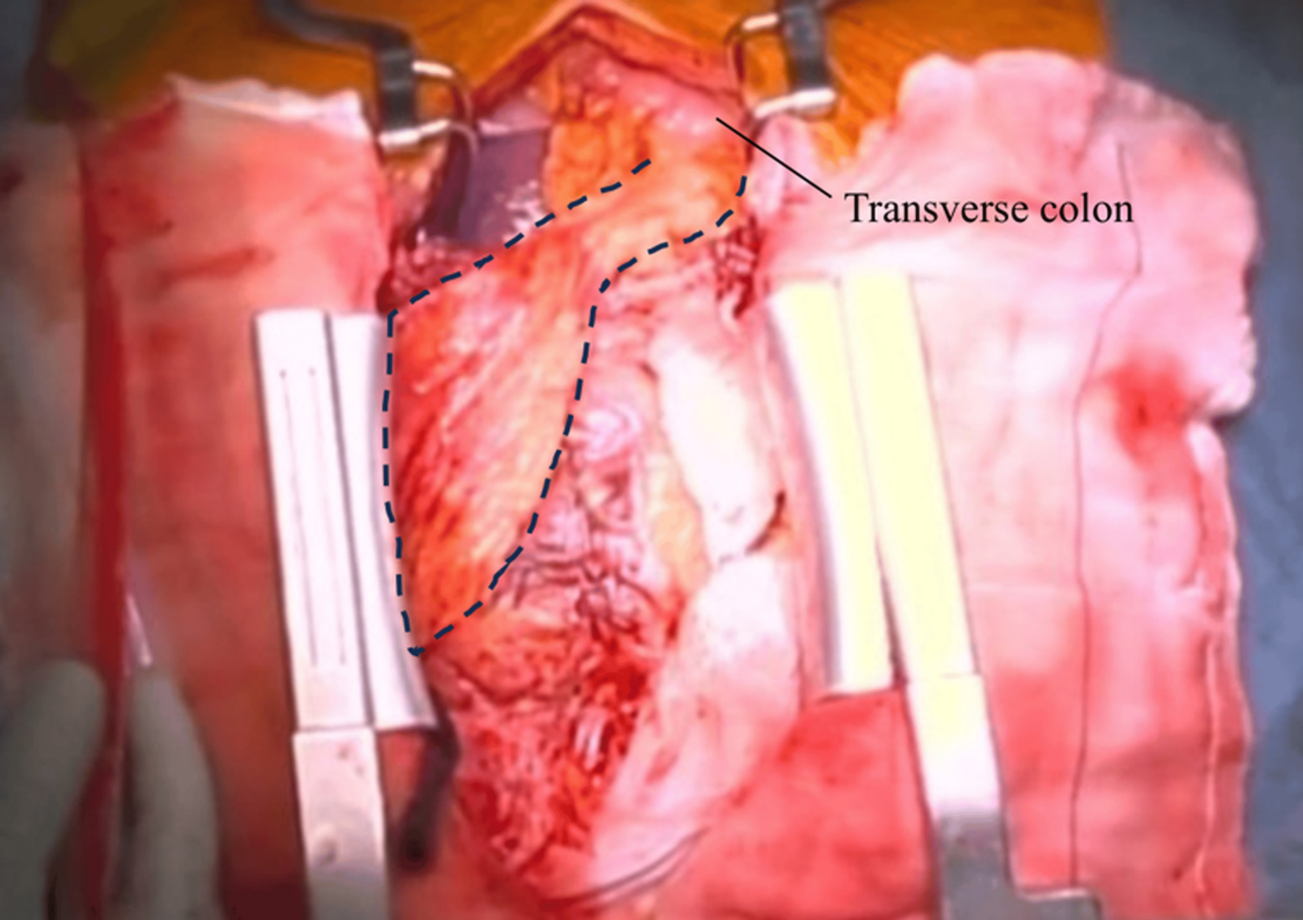
Intraoperative view The harvested omentum, with preserved right gastroepiploic artery flow (outlined by a black dotted line), was fixed to cover the left ventricle. The right side corresponds to the surgeon’s side, and the left side corresponds to the patient’s left.

However, the pericardial cavity was found to be densely adherent, and it was unclear whether all foreign materials had been completely removed. In light of this uncertainty, and to minimize the risk of recurrence, a pedicled omental flap based on the right gastroepiploic artery was harvested through a 6-cm subxiphoid abdominal incision. The flap was passed through a small diaphragmatic incision and transposed into the pericardial space, covering the left ventricular anterior wall (Figure 4).

The chest was closed using sternal wires. The patient was extubated eight hours postoperatively and had an uneventful recovery. Intravenous cefazolin (2 g, three times daily) was administered postoperatively but discontinued after one week, as both intraoperative cultures and histopathological examination supported a diagnosis of sterile inflammation. Histology revealed infiltration of neutrophils and lymphocytes without evidence of granulomatous inflammation or foreign body giant cells, and no microorganisms were identified. These findings were consistent with a chronic sterile inflammatory response, likely induced by retained hemostatic materials.

**Figure 5:**
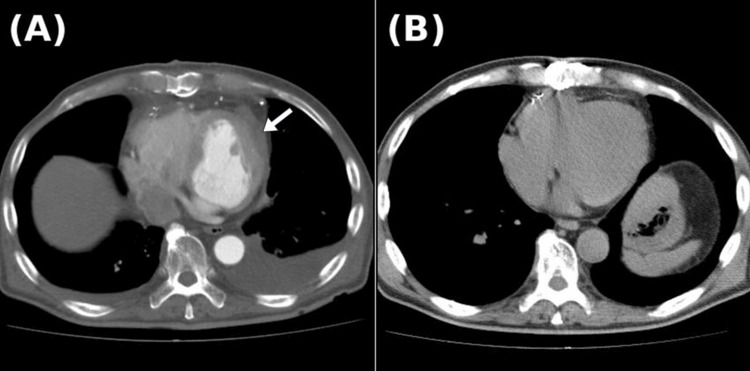
Postoperative computed tomography (CT) images (A) A small amount of fluid collection is seen at the site of foreign body removal during hospitalization (white arrow). (B) Follow-up computed tomography (CT) at six months postoperatively shows complete resolution of the fluid collection.

Postoperative CT during hospitalization revealed only minimal residual fluid (Figure 5A), which had completely resolved by the six-month outpatient follow-up (Figure 5B). The surgical site healed without complications, and the patient remained asymptomatic at subsequent visits.

## Discussion

This case represents a rare but clinically significant complication of cardiovascular surgery: a delayed sterile abscess secondary to retained hemostatic materials. TachoSil, Hydrofit, and Teflon felt are all effective in achieving immediate hemostasis in fragile or inaccessible cardiac tissue [1-3]. However, their long-term presence in the pericardial or mediastinal space may incite a chronic inflammatory response, resulting in fluid collection, tissue encapsulation, and, in some cases, fistulization to the overlying skin [4,5]. TachoSil is intended to undergo gradual bioresorption; however, in regions with limited vascularity or within encapsulated spaces, incomplete degradation may occur, potentially sustaining a localized immune response [7]. In contrast, Hydrofit and Teflon felt are non-absorbable materials that can persist indefinitely in situ and serve as a nidus for sterile inflammation or delayed infectious complications, even years following implantation [4,8].

The absence of systemic inflammatory markers and consistently negative cultures differentiated this case from a typical postoperative mediastinitis. This highlights the diagnostic dilemma posed by sterile abscesses, where clinical suspicion and imaging findings must guide decision-making. The CT findings of a low-density collection, communicating from the anterior chest wall to the pericardial space, were key in identifying the deep-seated origin of the process and associating it with prior surgical manipulation.

Management of sterile abscesses secondary to foreign material must be definitive. Conservative approaches, such as antibiotics and percutaneous drainage, while initially appealing, often fail due to the presence of non-degradable or partially degraded agents that serve as ongoing inflammatory stimuli [5]. Complete or maximal safe removal of the foreign body is crucial. In this patient, the necessity for cardiopulmonary bypass underscored the difficulty and risk of resternotomy in the setting of dense adhesions.

The use of a pedicled omental flap in this context is particularly noteworthy. The omentum is known for its angiogenic, immunomodulatory, and absorptive properties. It has been widely used in managing deep sternal wound infections and prosthetic graft infections, where its ability to obliterate dead space, promote neovascularization, and control inflammation proves invaluable [9-11]. In this case, dense adhesions within the pericardium made it impossible to confirm complete removal of foreign material. Therefore, omental transposition was employed not only for its therapeutic benefits but also as a preventive strategy against recurrence. This decision was retrospectively validated by postoperative imaging, which demonstrated complete resolution of the fluid collection. These findings strongly support the omental flap’s role in both healing and recurrence prevention.

Although the use of an omental flap offers significant therapeutic advantages in managing mediastinal complications, potential risks such as donor site herniation, flap necrosis, or infection have been reported [9-11]. However, the incidence of these complications remains low in contemporary series, and the overall benefits - including dead space obliteration, infection control, and immune modulation - generally outweigh the risks in selected patients.

This case highlights the potential for delayed complications related to retained topical hemostatic agents, as the sterile abscess manifested nearly two years after the initial surgery. While these agents provide unquestionable benefits in achieving acute hemostasis, their long-term consequences must not be overlooked - particularly in elderly or comorbid patients who may have impaired resorption or healing capacity. Non-absorbable or slow-degrading materials can persist and trigger chronic inflammatory responses, even after an initially uneventful recovery. Therefore, surgeons should remain vigilant during long-term follow-up and maintain a high index of suspicion when patients develop new localized symptoms near previous surgical sites.

## Conclusions

This case highlights the potential for delayed sterile abscess formation due to retained hemostatic materials used in cardiac surgery. It reinforces the importance of recognizing sterile inflammatory complications, the limitations of conservative management, and the effective use of omental flap transposition. Early surgical intervention remains the mainstay for definitive management of such cases, and awareness of this entity is crucial for timely diagnosis and successful treatment.
